# An open quantum system approach to the radical pair mechanism

**DOI:** 10.1038/s41598-018-34007-4

**Published:** 2018-10-24

**Authors:** Betony Adams, Ilya Sinayskiy, Francesco Petruccione

**Affiliations:** 1 Quantum Research Group, School of Chemistry and Physics, University of KwaZulu-Natal, Durban KwaZulu-Natal, 4001 South Africa; 2 National Institute for Theoretical Physics (NITheP), KwaZulu-Natal, 4001 South Africa; 3 School of Electrical Engineering, KAIST, Daejeon, 34141 Republic of Korea

## Abstract

The development of the radical pair mechanism has allowed for theoretical explanation of the fact that magnetic fields are observed to have an effect on chemical reactions. The mechanism describes how an external magnetic field can alter chemical yields by interacting with the spin state of a pair of radicals. In the field of quantum biology, there has been some interest in the application of the mechanism to biological systems. This paper takes an open quantum systems approach to a model of the radical pair mechanism in order to derive a master equation in the Born-Markov approximation for the case of two electrons, each interacting with an environment of nuclear spins as well as the external magnetic field, then placed in a dissipative bosonic bath. This model is used to investigate two different cases relating to radical pair dynamics. The first uses a collective coupling approach to simplify calculations for larger numbers of nuclei interacting with the radical pair. The second looks at the effects of different hyperfine configurations of the radical pair model, for instance the case in which one of the electrons interact with two nuclei with different hyperfine coupling constants. The results of these investigations are analysed to see if they offer any insights into the biological application of the radical pair mechanism in avian magnetoreception.

## Introduction

There is a long history of experimental evidence for magnetic field modulation of chemical reactions^1,2^. The concept of the radical pair mechanism, by which a magnetic field can change the dynamics of a chemical reaction, arose out of investigations made into the relevance of nuclear and electronic spin phenomena to chemical reactions, as long ago as the late 1960s^3^. Since its introduction as a means to explain such phenomena the theory has been well documented^3–6^. Recent interest in magnetic field effects and the radical pair mechanism include a variety of subjects. The emerging field of quantum biology in particular has been instrumental in raising interest in the application of the radical pair mechanism to biological systems. Application of radical pair theory to avian magnetoreception began not long after the initial formalisation of the mechanism. First proposed by Schulten *et al*. in the 1970s, there is a wealth of theoretical and experimental literature dedicated to the topic^1^. It has also been suggested that radical pairs in photosynthetic reaction centres play a number of different roles from polarisation to protection mechanisms^7,8^. The role played by cryptochromes in circadian rhythms is well established^9,10^. More recently the observation of radical pairs in the flavoprotein cryptochrome has led to speculation that the mechanism controlling the circadian clock in some organisms could likely be the radical pair mechanism^11–13^. There has also been some debate about the role of magnetic field effects in enzyme reactions. Flavin-dependent enzymes which are implicated in processes as diverse as energy production, apoptosis, DNA repair and neural development have been less successfully investigated with respect to magnetic field effects, while researchers are trying to replicate the report that a magnetic field can alter enzymatic ATP synthesis^14–16^. The research is possibly important in the context of our growing environmental exposure to electromagnetic fields and the lack of consensus on whether such weak fields might have an effect on biological organisms.

The radical pair can be described in the following rudimentary outline of the three important steps that constitute the mechanism. In the first step a photon is incident on the donor molecule of the pair, causing an electron to be excited and donated to the acceptor molecule. This results in a spin-correlated but spatially separated electron pair which is conventionally thought of as being in a singlet state. During the second step the radical pair oscillates between singlet and triplet state under the influence of the Zeeman or hyperfine interaction. The third step is then recombination of the pair to form some sort of chemical product/signal. The chemical product formed in this last step is dependent on whether the pair is in a singlet or triplet state, and is thus dependent on the magnetic field. This allows the radical pair to function as a compass.

This paper proposes an open quantum systems approach to the second step of the mechanism. Although the theoretical approach outlined in this paper is not restricted to the case of avian magnetoreception, it has been simulated using parameters relevant to this specific application. The results of these simulations have also been analysed to see if they offer any insights into biological systems, specifically the avian compass. To this end the paper presents two different investigations. The first of these offers a novel theoretical approach to the radical pair mechanism in the form of the collective coupling model. The second looks more closely at the hyperfine environment of the radical pair to see how altering hyperfine coupling strength might alter radical pair dynamics. The motivation for these investigations is noted briefly below.

In his 1976 paper Haberkorn describes in detail a density matrix treatment of radical pair reactions and most subsequent approaches in the field have since followed his lead, although there have been attempts to investigate the advantages and limitations of this approach^17–19^. In the context of avian migration, one of the limitations to the conventional approach is the increased computational complexity that arises as the radical pair interacts with increasing numbers of nuclei. It is estimated that the number of significant nuclei felt by each radical is approximately ten to fifteen^20–22^. As a means to simplify these calculations this paper outlines an approach in which the Hamiltonian is a function of the collective spin operators of the nuclear spin environment and the space of the total system can be decomposed as the sum of the subspaces^23–25^.

The collective model is useful for the purpose of simplification, to get a broad idea of the effects of increased numbers of nuclei and to look for potential patterns of radical pair behaviour. This simplification, however, is problematic in the context of the complex biological environment. To address this we adapted the model for the case of two non-identical nuclei having the predominant influence on radical pair dynamics, where by non-identical we specifically mean that the nuclei are not confined to having the exact same hyperfine coupling strength. We then investigated the effects of various arrangements of the nuclei interacting with the radical pair. There have been a number of reports detailing experimental results in which electromagnetic radiation disrupts the avian compass^26–28^. The most recent of these indicates that a broad band of radiofrequency radiation is responsible for this disruption^28^. The master equation derived in this paper is written in terms of the transition frequencies associated with the singlet-triplet oscillations in the radical pair. These transition frequencies depend on the magnetic field strength as well as the hyperfine coupling constants. By investigating different hyperfine parameters and the way in which they altered the radical pair dynamics we hoped to gain some insight into how such a broad band of electromagnetic radiation might interact with the radical pair.

## Results

In these investigations, both for the case of the collective model as well as the non-identical nuclei, we follow an open quantum systems master equation route where the Lindblad master equation derived is trace-preserving (preserves physical probabilities), but it differs from the conventional approach in two main respects. First, our master equation is not restricted to demonstrating only the effects of the weak interaction. Second, our model focuses only on the dynamics of the radical pair prior to and not including recombination. Our interest is in the coherence lifetime of the radical pair and the effects of different hyperfine environments on these dynamics. It should be clarified that the rates under scrutiny are thus not the rates at which recombination happens, or product is formed, but rather the rates governing singlet/triplet interconversion. Any further dynamics relating to production of singlet or triplet specific chemicals would still need to be added to the master equation.

### A model of the system

**Figure 1:**
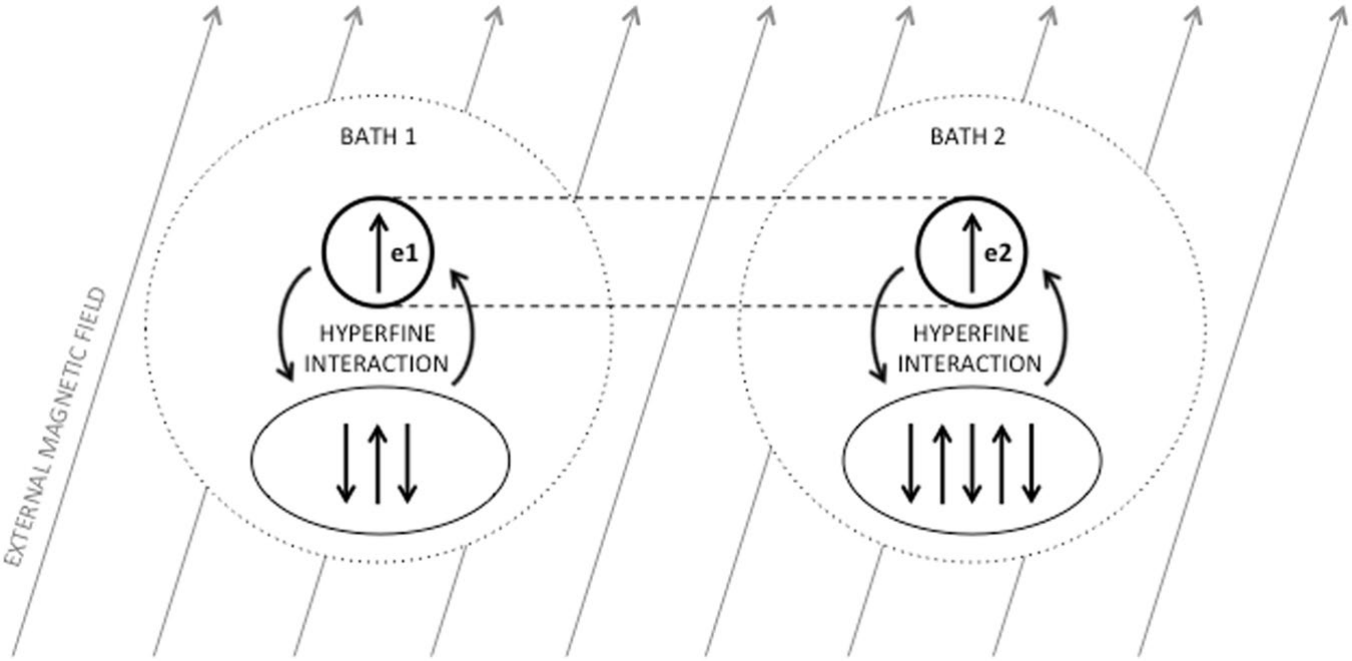
A diagram of the model shows the two electrons *e*_1_ and *e*_2_ that make up the spatially separated but spin correlated radical pair. Each electron experiences a hyperfine interaction with its nuclear environment, represented here by three and five arrows (nuclei) for *e*_1_ and *e*_2_ respectively. Both electrons experience the Zeeman effect due to the external geomagnetic field. Each of the radicals in the pair (modelled as the system, in Section (2.1)) is then assumed to interact with an external heat bath, undergoing decoherence and dissipation. This allows for a mathematical description of the second step of the radical pair mechanism, as outlined in the Introduction.

Consider the complete spin Hamiltonian for the radical pair^29^:

1 $$H={H}_{{\rm{zee}}}+{H}_{{\rm{hf}}}+{H}_{{\rm{dip}}}+{H}_{{\rm{ex}}}+{H}_{{\rm{nuc}}},$$

where *H*_zee_ and *H*_hf_ are the Zeeman and hyperfine effects and are the only two terms retained. The dipole and exchange effects, *H*_dip_ and *H*_ex_, can be discounted either due to sufficient separation of the electrons or because the separation is optimal for the effects to cancel^30,31^. *H*_nuc_, the interaction of nuclear spins with the external field, is also neglected as the gyromagnetic ratio is much smaller than in the electronic case^29^. The Hamiltonian for this system models the two electrons, labelled (1) and (2), each interacting separately with their nuclear environment, see Fig. 1. The Hilbert space of the system is given by ${ {\mathcal H} }_{s}={ {\mathcal H} }_{e}^{(1)}\otimes { {\mathcal H} }_{e}^{(2)}\otimes { {\mathcal H} }_{n}^{(1)}\otimes { {\mathcal H} }_{n}^{(2)}$ where ${ {\mathcal H} }_{e}$ refers to the Hilbert space of either electron while ${ {\mathcal H} }_{n}$ refers to the Hilbert space of the nuclear spin environment.

### The collective coupling approach: Identical nuclei

As nuclei are added, the increasing degrees of freedom of the nuclear spin environment make solving the model more complicated. To address this we follow the approach in which the Hamiltonian is a function of the collective spin operators of the nuclear spin environment for each of the electrons (for our purposes we assume that the two electrons of the radical pair can be treated separately). In order to finally investigate the dynamics of the radical pair itself, the partial trace over the nuclear spin degrees of freedom is carried out within the subspaces corresponding to the different values of the total angular momentum of the surrounding nuclei^23–25^.

A particle with spin $\frac{1}{2}$ has a state that can be described by the complex inner product space, ${{\mathbb{C}}}^{2}$. A basis of this space can be constructed using the eigenvectors {|1〉, |0〉} corresponding to eigenvalues ±$\frac{1}{2}$ of the *z*-component of the spin operator ${S}_{z}=\frac{1}{2}{\sigma }_{z}$, where *σ*_*z*_ is one of the Pauli operators. Generalising this, for our system of *N* spin-half nuclei the state space is given by ${{\mathbb{C}}}^{2\otimes N}$ and a basis ${\otimes }_{i}^{N}|{\epsilon }_{i}\rangle$, ${\epsilon }_{i}=1,0$ can be constructed from the eigenvectors of the collective spin operator *I*_*z*_, with $\overrightarrow{I}=\frac{1}{2}\,{\sum }_{i=1}^{N}\,{\overrightarrow{\sigma }}_{i}$^23,25^. In the case of collective interaction it is known that |*j*, *m*〉 is common to the operators *I*^2^ and *I*_*z*_, with corresponding eigenvalues *j*(*j* + 1) and *m*. For a system of *N* nuclei the quantisation of angular momentum dictates that for *N* even $0\le j\le \frac{N}{2}$ and *N* odd $\frac{1}{2}\le j\le \frac{N}{2}$, with −*j* ≤ *m* ≤ *j*. For different values of *j* the scalar product of the state vectors is zero and the space of the total system can be decomposed as the sum of the subspaces^23,25^,

2 $$\underset{i=1}{\overset{N}{\otimes }}{{\mathbb{C}}}_{i}^{2}\mathop{=}\limits^{{\rm{sym}}}\underset{j=0,\frac{1}{2}}{\overset{\frac{N}{2}}{\otimes }}\nu (N,j){{\mathbb{C}}}^{2j+1},$$

where *ν*(*N*, *j*) is the degeneracy corresponding to a specific value of *j* and is given by

3 $$\begin{array}{rcl}\nu (N,j) & = & (\begin{array}{c}N\\ \frac{N}{2}-j\end{array})-(\begin{array}{c}N\\ \frac{N}{2}-j-1\end{array})\\  & = & \frac{2j+1}{\frac{N}{2}+j+1}\frac{N!}{(\frac{N}{2}-j)!(\frac{N}{2}+j)!}.\end{array}$$

The Hamiltonian for this system can now be written, with $\hslash =1$, as

4 $${H}_{s}={\gamma }_{e}(\overrightarrow{B}.{{\bf{S}}}^{(1)}+\overrightarrow{B}.{{\bf{S}}}^{(2)})+{\lambda }_{h}\,\sum _{k=1}^{2}\,\sum _{n,l=1}^{3}\,{A}_{nl}^{(k)}{{\bf{S}}}_{n}^{(k)}.{{\bf{I}}}_{l}^{(k)},$$

where $\overrightarrow{B}$ is the magnetic field vector. It is already taken into account that, for example, ${S}_{z}^{(1)}={S}_{z}^{(1)}\otimes$ ${{\mathbb{I}}}_{e}^{(2)}\otimes {{\mathbb{I}}}_{n}^{(1)}\otimes {{\mathbb{I}}}_{n}^{(2)}$ where ${{\mathbb{I}}}_{e}$ and ${{\mathbb{I}}}_{n}$ are the identity matrices for electron and nuclei. Thus an expression such as ${S}_{+}^{(1)}{I}_{-}^{(1)}$ uses ordinary matrix multiplication. **S** = (*S*_*x*_, *S*_*y*_, *S*_*z*_) is the vector of spin operators for each electron with ${S}_{x}=\frac{1}{2}{\sigma }_{x}$, ${S}_{y}=\frac{1}{2}{\sigma }_{y}$, ${S}_{z}=\frac{1}{2}{\sigma }_{z}$ and *σ*_*x*_, *σ*_*y*_, *σ*_*z*_ the Pauli matrices

$${\sigma }_{x}=(\begin{array}{cc}0 & 1\\ 1 & 0\end{array})\,{\sigma }_{y}=(\begin{array}{cc}0 & -\,i\\ i & 0\end{array})\,{\sigma }_{z}=(\begin{array}{cc}1 & 0\\ 0 & -\,1\end{array}).$$

**I** = (*I*_*x*_, *I*_*y*_, *I*_*z*_) is the vector of spin operators for nuclear spin.

The Zeeman interaction is represented by ${\gamma }_{e}\overrightarrow{B}.{\bf{S}}$ with electron gyromagnetic ratio *γ*_*e*_ = −*gμ*_*B*_, where *g* = 2 and *μ*_*B*_ is the Bohr magneton. The sum runs over *k* to include both electrons. The hyperfine coupling, as described by the hyperfine coupling tensor, must be anisotropic for the radical pair to be sensitive to different magnetic field alignments. Following the convention of quantum biology and the approach of ^32^ we assume the simplest case in which the first electron is anisotropically coupled to its spin environment while the second is isotropically coupled, with respective hyperfine coupling tensors

$${A}^{(1)}=(\begin{array}{ccc}2 & 0 & 0\\ 0 & 2 & 0\\ 0 & 0 & 1\end{array}),\,{A}^{(2)}=(\begin{array}{ccc}1 & 0 & 0\\ 0 & 1 & 0\\ 0 & 0 & 1\end{array})$$

and hyperfine coupling constant given by *λ*_*h*_.

The diagonalised system Hamiltonian is given by

5 $$\begin{array}{rcl}{H}_{S} & = & ({\gamma }_{e}{B}_{0}+{\lambda }_{h}\,j)|0,j,j\rangle \,\langle 0,j,j|-({\gamma }_{e}{B}_{0}-{\lambda }_{h}\,j)|1,j,-\,j\rangle \,\langle 1,j,-\,j|\\  &  & +\,\sum _{m=-j+1}^{j}\,[{v}_{1}(j,m)|{\lambda }_{jm}^{-}\rangle \,\langle {\lambda }_{jm}^{-}|+{v}_{2}(j,m)|{\lambda }_{jm}^{+}\rangle \,\langle {\lambda }_{jm}^{+}|]\\  &  & +\,\sum _{m=-j+1}^{j}\,[{u}_{1}(j,m)|{\varphi }_{jm}^{-}\rangle \,\langle {\varphi }_{jm}^{-}|+{u}_{2}(j,m)|{\varphi }_{jm}^{+}\rangle \,\langle {\varphi }_{jm}^{+}|].\end{array}$$

For details of this diagonalisation and the parameters involved see Supplementary information. The Born-Markov master equation for this system looks like

6 $$\begin{array}{rcl}\frac{d}{dt}{\rho }_{I}^{S}(t) & = & \sum _{k=1}^{2}\,{\gamma }_{D}({V}_{0}^{(k)}{\rho }_{I}^{S}(t){V}_{0}^{(k)}-\frac{1}{2}\{{V}_{0}^{(k)}{V}_{0}^{(k)},{\rho }_{I}^{S}(t)\})\\  &  & +\,\sum _{k=1}^{2}\,\sum _{q=1}^{{N}_{T}}\,{\gamma }_{q}(N({\omega }_{q}^{T})+1)\,({V}_{q}^{(k)}{\rho }_{I}^{S}(t){V}_{q}^{\dagger (k)}-\frac{1}{2}\{{V}_{q}^{\dagger (k)}{V}_{q}^{(k)},{\rho }_{I}^{S}(t)\})\\  &  & +\,{\gamma }_{q}N({\omega }_{q}^{T})\,({V}_{q}^{\dagger (k)}{\rho }_{I}^{S}(t){V}_{q}^{(k)}-\frac{1}{2}\{{V}_{q}^{(k)}{V}_{q}^{\dagger (k)},{\rho }_{I}^{S}(t)\}),\end{array}$$

where the *V*_*q*_ represent the transition operators, more details of which can be found in the Methods section below. The first term describes the effects of decoherence and the remaining terms describe the dissipation in the system. The sum runs over *k* = 2 to include both electrons, *N*_*T*_ is the number of transition operators, *γ*_*D*_ and *γ*_*q*_ are decoherence and dissipation rates as discussed below, and the Planck distribution

$$N({\omega }_{q}^{T})=\frac{1}{{e}^{\beta {\omega }_{q}^{T}}-1},$$

with $\beta =\frac{1}{{k}_{B}T}$, gives the number of thermal photons (bosons) in a mode of frequency ${\omega }_{q}^{T}$ at a given temperature *T* where *k*_*B*_ is the Boltzmann constant.

### Parameters

In order to investigate the biologically relevant dynamics of the radical pair as modelled in this paper we used parameters associated with the specific case of avian magnetoreception and we assumed the Zeeman effect to be that resulting from a geomagnetic field of 47 *μ*T^33^. In the relevant literature hyperfine coupling constants for organic molecules range in value. We selected across the range of kHz to MHz^21^ for the purposes of this paper. To accurately apply the model to biological systems we took the temperature to be 300 K. To calculate the appropriate frequency-dependent rates of dissipation we used

7 $${\gamma }_{q}=\frac{{\omega }_{q}^{3}}{3{\epsilon }_{0}\pi \hslash {c}^{3}}|d{|}^{2},$$

where *d* is the transition dipole moment. For details of this see the Supplementary information. With the parameters chosen for the magnetic field and hyperfine coupling constant the transition frequencies *ω*_*q*_ range from ≈10^5^–10^8^ Hz. For this range of transition frequencies a range of transition rates from 10^−9^–10^−3^ s^−1^ were calculated. The decoherence rate was taken to be at least ten times greater than the largest of the dissipation rates. While it is generally accepted that spin relaxation via spontaneous emission is very slow this is balanced in the model by the inclusion of relaxation due to thermal fluctuations, as represented by $N({\omega }_{q}^{T})$. Electron spins in radicals are relaxed by a number of other mechanisms, which occur faster than spontaneous emission. Typically spin relaxation results from local magnetic field fluctuations due to motion of the radical or its surroundings^34^. Lau *et al*. discuss the effects of rotational motion on the spin relaxation rate, demonstrating that the maximum relaxation rate can be up 100 times faster than the radical pair recombination^35^. The model described in this paper could be extended to consider such other mechanisms of spin relaxation through the inclusion of different spectral densities in the master equation.

### Results: collective coupling case

**Figure 2:**
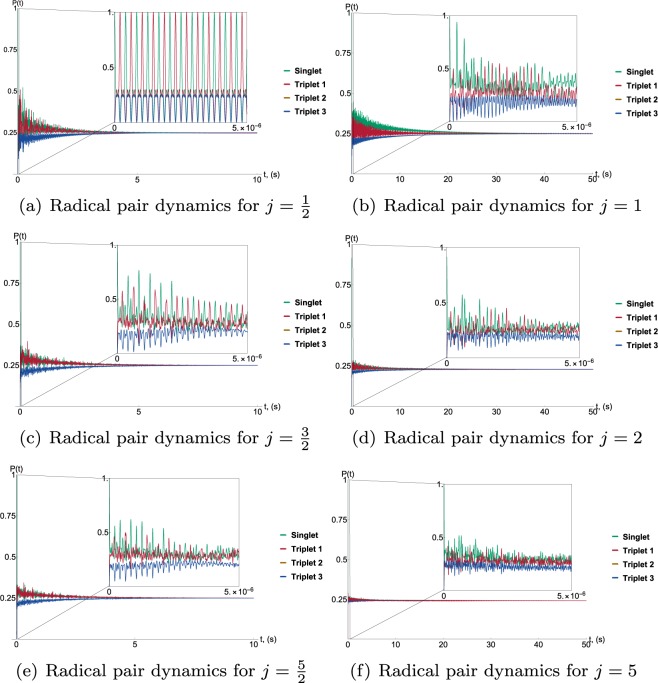
Graphs (**a**–**f**) illustrate the effects of decoherence and dissipation as the number of nuclei interacting with the radical pair increases. These effects are represented as the probability over time of finding the radical pair in one of the four possible spin states. There is a general trend towards a decreased coherence lifetime, this is because increasing the number of nuclei in the model, increases the number of possible transitions which allows for a greater dissipative effect from the environment. This effect is however not equal for odd and even numbers of nuclei, the radical pair decays slower when *j* is equal to integer values. The reason for this is that for integer values of *j* the possibility exists that both radicals in the pair could have *j* = 0 which leads to a particularly long lifetime. This is illustrated in Fig. 3. An additional difference between integer and non-integer *j* is the slightly faster oscillation between singlet and triplet states for the case of integer spin.

**Figure 3:**
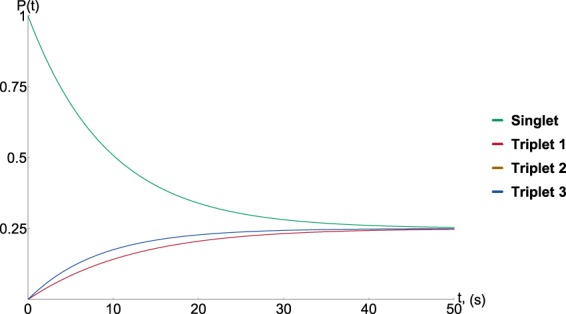
The graph illustrates the long lifetime for the case in which *j* = 0 for both radicals in the pair.

The collective coupling model is useful in that it allows for increased numbers of nuclei without overcomplicating the matrix calculations. In order to demonstrate any pattern that might emerge through the addition of nuclei we present each result, from a single nuclear interaction up to 10 nuclei. All results are represented as the probability of finding the radical pair in either of the four possible spin states at varying times. Triplet 1, 2 and 3 refer to the eigenstates of the operator for the *z*-component of the total electron spin, with the *z*-axis aligned with the magnetic field. While it can be seen that there is a general trend towards a decreased lifetime as the number of nuclei increases, see Fig. 2, Graphs (a)–(f), the effect is not equal for odd and even numbers of nuclei. An even number of nuclei, that is an integer value of *j*, results in slower decay of the singlet–triplet coherence and a longer pair lifetime. For integer values of *j*, *j* = 0 contributes to the sum of the subspaces that constitute the space of the total system, thus increasing the overall lifetime of the radical pair. This is illustrated by Fig. 3. An additional difference between odd and even cases can be seen in the shapes of the graphs as given by the inset details in Fig. 2. For integer values of *j* the singlet-triplet oscillation appears to happen slightly more quickly than it does for half-integer values of *j*.

The model predicts a coherence lifetime of the order of seconds, which seems surprisingly long-lived. It should be noted however that as nuclei are added this lifetime decreases, getting closer to the millisecond range. If this model were to be viewed in the context of biological systems, recent transient absorption measurements involving the avian compass candidate, cryptochrome, indicate radical pairs with millisecond lifetimes^33,36^. Although it should also be noted here that it is difficult to compare the lifetimes discussed in the literature due to the different interpretation of lifetime in this paper. It is used here to describe the persistence of the coherence whereas elsewhere it is used to describe how long the radicals last before they recombine.

It could be the case that the disparity between integer and half integer spins arises from the simplifications inherent in the model itself, which treats all the nuclei for each radical as identical spin-$\frac{1}{2}$ nuclei with identical hyperfine interactions, and would no longer be relevant should we consider a more realistic nuclear environment in which all hyperfine tensors are different and both spin-$\frac{1}{2}$ and spin-1 nuclei are present. However, the efficacy of the model is due exactly to this treatment of the nuclei as identical, which allowed us to simplify the increasing degrees of freedom for increasing numbers of nuclei by using the fact that the space of the total system could be decomposed as the sum of the subspaces. While this allowed us to identify any patterns that might emerge for increasing numbers of nuclei, the results presented next abandon the collective coupling approach and adapt the model to the slightly more realistic case in which the radical pair interacts with its nuclear environment with a range of hyperfine coupling strengths.

### Non-collective case

In this example we have investigated the effects of two different nuclei interacting anisotropically with one of the radicals in the pair while the other radical experiences no hyperfine interaction. Discounting all other interactions but the Zeeman and hyperfine effects, the system Hamiltonian can be written as

8 $${H}_{s}={\gamma }_{e}(\overrightarrow{B}.{{\bf{S}}}^{(1)}+\overrightarrow{B}.{{\bf{S}}}^{(2)})+{\lambda }_{h}\,\sum _{n=1}^{2}\,\sum _{i=x,y,z}\,{\omega }_{i}^{(n)}{{\bf{S}}}_{i}^{(n)}\otimes {{\bf{I}}}_{i}^{(n)},$$

where $\overrightarrow{B}$ is the magnetic field vector and the second electron has no hyperfine interaction. The sum over n for the first electron represents the two different nuclei and the *ω*_*i*_ represent the anisotropic hyperfine coupling strengths. Once again **S** = (*S*_*x*_, *S*_*y*_, *S*_*z*_) is the vector of spin operators for each electron with ${S}_{x}=\frac{1}{2}{\sigma }_{x}$, ${S}_{y}=\frac{1}{2}{\sigma }_{y}$, ${S}_{z}=\frac{1}{2}{\sigma }_{z}$ and *σ*_*x*_, *σ*_*y*_, *σ*_*z*_ the Pauli matrices

$${\sigma }_{x}=(\begin{array}{cc}0 & 1\\ 1 & 0\end{array})\,{\sigma }_{y}=(\begin{array}{cc}0 & -\,i\\ i & 0\end{array})\,{\sigma }_{z}=(\begin{array}{cc}1 & 0\\ 0 & -\,1\end{array}).$$

**I** = (*I*_*x*_, *I*_*y*_, *I*_*z*_) is the vector of spin operators for nuclear spin.

The Zeeman interaction is represented by ${\gamma }_{e}\overrightarrow{B}\mathrm{.}{\bf{S}}$ with electron gyromagnetic ratio *γ*_*e*_ = −*gμ*_*B*_, where *g* = 2 and *μ*_*B*_ is the Bohr magneton. The system Hamiltonian for the non-collective case is diagonalised by finding eigenvectors and eigenvalues. The master equation in this case is then of the same form as Equation (6), however with distinctive transition operators relevant to the system in question.

### Results: Non-identical nuclei

While the collective model demonstrates some potentially interesting differences between integer and non-integer collective spins it still falls short as a realistic model as might be applied to complex biological systems, most obviously in the fact that it uses a single coupling strength for each nucleus. A more appropriate approach in this case would be to allow for a number of nuclei with differing coupling strengths to interact with the radical pair. We present the results of this approach below.

As in the case of the collective coupling instance we employed parameters relevant to biological systems to simulate the master equation. Temperature was taken to be 300 K, the magnetic field 47 *μ*T^33^ and organic hyperfine coupling constants in the range of kHz to MHz. Due to the fact that two different hyperfine coupling constants are used there is a greater range of transition frequencies and corresponding transition rates.

**Figure 4:**
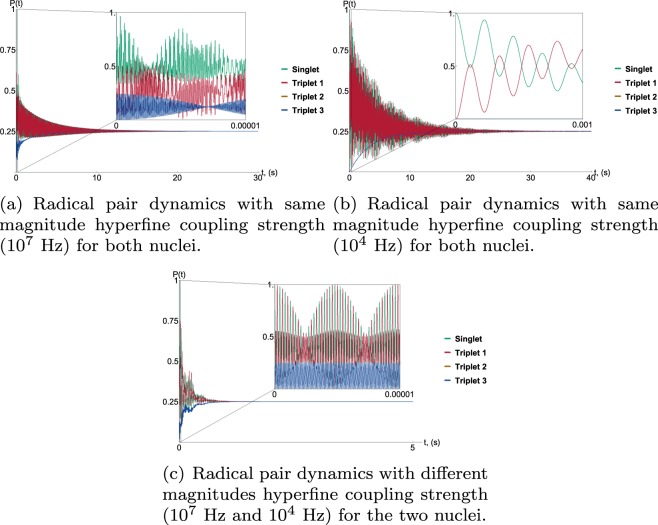
Graphs (**a**–**c**) illustrate the effects that different configurations of hyperfine coupling strength have on the rate of decoherence and dissipation for the radical pair. What is interesting is that the strength of the coupling constant does not dominantly influence the coherence lifetime of the radical pair. Where both nuclei interact with comparable magnitude (both of the order of 10^7^ for Graph (**a**) and both of the order of 10^4^ for Graph (**b**)) the dissipation happens more slowly whereas if the two nuclei have markedly different coupling magnitudes (Graph (**c**)) the dissipation happens more quickly. This is because in the latter configuration a greater proportion of the transition frequencies give rise to quicker transition rates. This suggests a less than straightforward correlation between hyperfine coupling strength and coherence lifetime, where there might be an optimal combination of hyperfine coupling constants that allows for longer lasting coherence. The inset details of Graphs (**a**–**c**) demonstrate a number of different dynamics. Where the size of the coupling constant does have an effect is in the rate of oscillation between singlet and triplet states. This is clearly illustrated by the fast oscillations of Graph (**a** and **c**), where at least one of the nuclei has a strong coupling constant, while Graph (**b**), where both nuclei interact with a comparable but smaller magnitude (10^4^ as opposed to 10^7^ Hz) the singlet and triplet 1 states oscillate very slowly and triplet states 2 and 3 do not oscillate at all. Note the different time scale. Finally, the ‘beating’ effect, or additional slower oscillation, that is apparent in Graphs (**a** and **c**) arises from the Zeeman contribution of the magnetic field where the field strength (1.3 × 10^6^ Hz) is comparable to the strength of at least one of the hyperfine coupling strengths.

We investigated three different hyperfine configurations with the two nuclei interacting anisotropically with one of the electrons in the pair. In the first case, both nuclei had a comparable hyperfine coupling strength of the order of 10^7^ Hz. In the second, the two nuclei again had a comparable but much smaller hyperfine coupling of the order of 10^4^ Hz. In the third case, the hyperfine coupling strengths of the two nuclei differed, one nucleus had a hyperfine coupling constant of 10^7^ Hz with the other set at 10^4^ Hz. Although it might be expected that dissipation would be enhanced by larger hyperfine coupling constants, the results show otherwise and are presented in Fig. 4. The coherence lifetime is comparably longer for both cases in which the nuclei had similar hyperfine coupling magnitudes (Graph (a) and Graph (b)) independent of whether these coupling constants were both 10^7^ Hz or both 10^4^ Hz (although it is slightly shorter for the case in which the coupling constants are both large). The coherence lifetime is much shorter, however, if the two nuclei each had a very different coupling strength (Graph (c)). This is because the transition frequencies depend on various combinations of the hyperfine coupling constants and the magnetic field strength. For the case in which both nuclei have a different magnitude coupling constant this gives rise to a greater proportion of transition frequencies with correspondingly fast transition rates. It is perhaps possible to conclude, therefore, that the coherence lifetime is sensitive to a nuclear environment that is varied rather than being solely dependent on the strength of the hyperfine coupling.

Where the magnitude of the coupling constants is important, however, is with respect to the rate of singlet-triplet oscillation. It can be seen from the inset in Graph (b) that when both coupling constants are too small the singlet and triplet 1 states oscillate very slowly and the triplet 2 and triplet 3 states do not oscillate at all. This would be important to note should this model be applied to, for example, the avian compass. If the radical pair of avian magnetoreception has a recombination lifetime of much smaller than milliseconds then the singlet would not have time to oscillate at all. Thus the magnitude of the coupling constants is important for the functioning of the radical pair. The model further demonstrates that it is sufficient to have one nucleus with larger coupling constant for the oscillation to be ensured (Graph (c) inset).

## Methods

### The master equation

If the Hamiltonian of a quantum system is the sum of a free term and an interaction term

9 $$H={H}_{0}+{H}_{I}$$

then it is useful to describe the dynamics in the interaction picture, in which both state vectors and operators evolve in time. In the chosen model the system constituted by the radical pair in its nuclear environment is placed in a bath and allowed to dissipate. The bath Hamiltonian is given as

10 $${H}_{B}=\sum _{k=1}^{2}\,\sum _{{n}_{k}}\,{\omega }_{{n}_{k}}{a}_{{n}_{k}}^{\dagger }{a}_{{n}_{k}},$$

where the *ω*_*n*_ are the frequencies of the *n*–th bosonic operator, ${a}_{n}^{\dagger }$ is the creation operator and *a*_*n*_ is the annihilation operator. As the evolution of the system is happening at physiological temperatures it is natural to assume the system is embedded in a dissipative bosonic environment^37^. The interaction Hamiltonian can be written as

11 $${H}_{I}=\sum _{k=1}^{2}\,\sum _{{n}_{k}}\,[{g}_{{n}_{k}}{a}_{{n}_{k}}+{\bar{g}}_{{n}_{k}}{a}_{{n}_{k}}^{\dagger }]\otimes [\alpha ({S}_{x}^{(k)}+{S}_{z}^{(k)})],$$

with *α* ≥ 0 a model parameter weighting the extent to which *S*_*x*_ and *S*_*z*_ contribute. Typically in the theory of open quantum systems one would use *S*_*x*_ or *S*_*z*_ coupling only, representing either dissipation or decoherence. As there is currently no evidence which is the dominant interaction both are included equally here. In order to proceed with the formulation of the interaction Hamiltonian it is necessary to calculate the transition operators specific to the system.

### The transition operators

The transition operators result from the decomposition of the operator *V* = *S*_*x*_ + *S*_*z*_ in the basis of the eigenoperators of the system Hamiltonian *H*_*S*_^38^. They are found using

$$[{H}_{S}^{(1)},{V}_{q}]=-\,{\omega }_{q}{V}_{q}\,{\rm{and}}\,[{H}_{S}^{(1)},{V}_{q}^{\dagger }]={\omega }_{q}{V}_{q}^{\dagger },$$

where *q* here labels the number of transition operators and the transition frequencies corresponding to each operator, *ω*_*q*_ ≥ 0, are expressed in terms of the magnetic field and the hyperfine coupling constant. A transition frequency equal to zero corresponds to decoherence in the system, which is found using

$$[{H}_{S}^{(1)},{V}_{0}]=0.$$

Consider, for example in the case of collective coupling, a single nucleus interacting with the first electron, $j=\frac{1}{2}$. In this instance there are five transition operators, as given in Table 1 with corresponding transition frequencies. The decoherence in this case is given by

12 $$\begin{array}{rcl}{V}_{0} & = & \frac{1}{2}|0,j,j\rangle \,\langle 0,j,j|+\frac{1}{2}\,\cos \,2\theta |{\lambda }_{jj}^{-}\rangle \,\langle {\lambda }_{jj}^{-}|-\frac{1}{2}\,\cos \,2\theta |{\lambda }_{jj}^{+}\rangle \,\langle {\lambda }_{jj}^{+}|\\  &  & -\,\frac{1}{2}|1,j,-\,j\rangle \,\langle 1,j,-\,j|.\end{array}$$

**Table 1:** Example transition operators for $j=\frac{1}{2}$.

	Transition operator	Transition frequency
*V*_1_	$\frac{\cos \,\theta }{2}|0,j,j\rangle \,\langle {\lambda }_{jj}^{+}|$	$\frac{1}{2}({\gamma }_{e}{B}_{0}+{\lambda }_{h}\,j)-{v}_{2}(j,j)$
*V*_2_	$-\,\frac{\sin \,\theta }{2}|{\lambda }_{jj}^{-}\rangle \,\langle 0,j,j|$	$-\,\frac{1}{2}({\gamma }_{e}{B}_{0}+{\lambda }_{h}\,j)+{v}_{1}(j,j)$
*V*_3_	$\frac{\cos \,\theta }{2}|{\lambda }_{jj}^{-}\rangle \langle 1,j,-\,j|$	$-\,\frac{1}{2}({\gamma }_{e}{B}_{0}-{\lambda }_{h}\,j)+{v}_{1}(j,j)$
*V*_4_	$\frac{\sin \,\theta }{2}|1,j,-\,j\rangle \,\langle {\lambda }_{jj}^{+}|$	$-\,\frac{1}{2}({\gamma }_{e}{B}_{0}-{\lambda }_{h}\,j)-{v}_{2}(j,j)$
*V*_5_	$\frac{\cos \,\theta \,\sin \,\theta }{2}|{\lambda }_{jj}^{-}\rangle \,\langle {\lambda }_{jj}^{+}|$	*v*1(*j*, *j*) − *v*2(*j*, *j*)
${V}_{1}^{\dagger }$	$-\,\frac{\sin \,\theta }{2}|0,j,j\rangle \,\langle {\lambda }_{jj}^{-}|$	$\frac{1}{2}({\gamma }_{e}{B}_{0}+{\lambda }_{h}\,j)-{v}_{1}(j,j)$
${V}_{2}^{\dagger }$	$\frac{\cos \,\theta }{2}|{\lambda }_{jj}^{+}\rangle \,\langle 0,j,j|$	$-\,\frac{1}{2}({\gamma }_{e}{B}_{0}+{\lambda }_{h}\,j)+{v}_{2}(j,j)$
${V}_{3}^{\dagger }$	$\frac{\sin \,\theta }{2}|{\lambda }_{jj}^{+}\rangle \langle 1,j,-\,j|$	$\frac{1}{2}({\gamma }_{e}{B}_{0}-{\lambda }_{h}\,j)+{v}_{2}(j,j)$
${V}_{4}^{\dagger }$	$\frac{\cos \,\theta }{2}|1,j,-\,j\rangle \,\langle {\lambda }_{jj}^{-}|$	$-\,\frac{1}{2}({\gamma }_{e}{B}_{0}-{\lambda }_{h}\,j)-{v}_{1}(j,j)$
${V}_{5}^{\dagger }$	$\frac{\cos \,\theta \,\sin \,\theta }{2}|{\lambda }_{jj}^{+}\rangle \,\langle {\lambda }_{jj}^{-}|$	−*v*_1_(*j*, *j*) + *v*_2_(*j*, *j*)

Transition operators for the non-collective case are found in a similar manner. Using the transition operators the interaction Hamiltonian for one electron can now be written as

13 $${H}_{I}=\sum _{n}\,[{g}_{n}{a}_{n}{e}^{-i{\omega }_{n}t}+{\bar{g}}_{n}{a}_{n}^{\dagger }{e}^{i{\omega }_{n}t}]\otimes [\sum _{q=1}^{{N}_{T}}\,({V}_{q}{e}^{-i{\omega }_{q}^{T}t}+{V}_{q}^{\dagger }{e}^{i{\omega }_{q}^{T}t})+{V}_{0}],$$

where *N*_*T*_ is the number of transition operators for the particular system. By applying the rotating wave and Born-Markov approximations the interaction master equation for both electrons looks like

14 $$\begin{array}{rcl}\tfrac{d}{dt}{\rho }_{I}^{S}(t) & = & \sum _{k=1}^{2}\,{\gamma }_{D}({V}_{0}^{(k)}{\rho }_{I}^{S}(t){V}_{0}^{(k)}-\tfrac{1}{2}\{{V}_{0}^{(k)}{V}_{0}^{(k)},{\rho }_{I}^{S}(t)\})\\  &  & +\,\sum _{k=1}^{2}\,\sum _{q=1}^{{N}_{T}}\,{\gamma }_{q}(N({\omega }_{q}^{T})+1)\,({V}_{q}^{(k)}{\rho }_{I}^{S}(t){V}_{q}^{\dagger (k)}-\tfrac{1}{2}\{{V}_{q}^{\dagger (k)}{V}_{q}^{(k)},{\rho }_{I}^{S}(t)\})\\  &  & +\,{\gamma }_{q}N({\omega }_{q}^{T})\,({V}_{q}^{\dagger (k)}{\rho }_{I}^{S}(t){V}_{q}^{(k)}-\tfrac{1}{2}\{{V}_{q}^{(k)}{V}_{q}^{\dagger (k)},{\rho }_{I}^{S}(t)\}),\end{array}$$

where the first term describes the effects of decoherence and the remaining terms describe the dissipation in the system. The sum runs over *k* = 2 to include both electrons, *N*_*T*_ is the number of transition operators, *γ*_*D*_ and *γ*_*q*_ are decoherence and dissipation rates as discussed above, and the Planck distribution

$$N({\omega }_{q}^{T})=\frac{1}{{e}^{\beta {\omega }_{q}^{T}}-1},$$

with $\beta =\frac{1}{{k}_{B}T}$, gives the number of thermal photons (bosons) in a mode of frequency ${\omega }_{q}^{T}$ at a given temperature *T* where *k*_*B*_ is the Boltzmann constant.

## Discussion

The open quantum systems approach to the radical pair mechanism presented in this paper demonstrated a novel theoretical approach (the collective coupling approach) as well as a novel numerical result (variation rather than hyperfine coupling strength magnifies dissipative effects). This allows some tentative conclusions to be made with respect to the radical pair mechanism. First, the collective coupling simplified calculations for increasing numbers of nuclei and showed that when the radicals in the pair interact with even numbers of nuclei the coherence lifetime of the pair is extended. This suggests that for a hyperfine environment consisting of identically coupled nuclei, the radical pair coherence might be preserved by the case in which an even number of nuclei give the dominant hyperfine interaction. Second, the results of the non-collective approach suggest that the coherence lifetime does not simply depend on the magnitude of the hyperfine coupling constant but rather on a combination of coupling strengths which might be optimised to offset dissipation and extend the coherence lifetime.

As applied to the biological context, one of the advantages of the model is that the master equation is formulated in terms of transition frequencies. If the model is to be applied to specific biological systems then it should be tailored to address the experimental evidence associated with these systems. For instance, avian magnetoreception has been postulated to rely on the radical pair mechanism and it has been demonstrated that birds are disoriented in oscillating radiofrequency fields^26–28^. This would be the case if the frequency of the applied field corresponded to one of the various energy-level splittings of the hyperfine interaction. The model outlined in this paper could be used in an attempt to reproduce these results, to see what specific effects these resonances might have on the dynamics of the radical pair. The most recent of these studies introduced two non-overlapping frequency bands from 20 kHz to 450 kHz or from 600 kHz to 3 MHz^28^ and demonstrated the birds’ disorientation under both, thus proving that this effect is unlikely to be the result of a single specific frequency or confined to one part of the radio frequency spectrum. That such a broad range of frequencies could cause resonance effects is already suggested by the model of the radical pair developed in this paper. As discussed previously the transition frequencies follow from the external magnetic field and the hyperfine coupling constant. For example, ${\omega }_{q}^{T}={\gamma }_{e}{B}_{0}+{\lambda }_{h}\,j$. Choosing the hyperfine coupling constant to be *λ*_*h*_ = 30 MHz, resulted in transition frequencies ranging from 1–100 MHz. In addition to this, if, as it is estimated, the radical pair interacts with 10 nuclei, this would give 50 different transition frequencies across a range from kHz to MHz. This is the case if a single value for *λ*_*h*_ is chosen. If it is taken into consideration that each radical might interact with each of these 10 nuclei with differing strengths and a range of *λ*_*h*_ then the number of possible transition frequencies runs into the hundreds. Thus it might be conceivable that the complexity of the hyperfine environment does allow for effects across such a broad range of frequencies.

If it conceivable that birds employ quantum effects through the radical pair mechanism in something as integral to their survival as migration then it is not too much of a leap to consider that other complex species might also make use of radical pairs. it has recently been shown that the molecule in which the radical pair mechanism manifests in birds is the flavoprotein cryptochrome^39,40^. Cryptochrome is also found in the human retina^41^ and it stands to reason that it might be employed through a mechanism similar to that of avian cryptochrome. It would thus be extremely useful to develop a model by which this mechanism might be investigated, a model that is both simple enough to simulate for differing hyperfine environments but not so simple as to lose biological relevance. It is to this end that this paper is pointed.

## Electronic supplementary material

Supplementary Information
