# Supplementary material for: An open quantum system approach to the radical pair mechanism
Source: Sci Rep. 2018 Oct 24;8:15719. doi: 10.1038/s41598-018-34007-4 (PMC6200754; doi:10.1038/s41598-018-34007-4)
Supplement: Supplementary file 1 — Supplementary Information [file 41598_2018_34007_MOESM1_ESM.pdf]

# An open quantum system approach to the radical pair mechanism

**Betony Adams**[1,2,*], **Ilya Sinayskiy**[1,2] **and Francesco Petruccione**[1,2,3]

[1] Quantum Research Group, School of Chemistry and Physics, University of KwaZulu-Natal, Durban, KwaZulu-Natal, 4001, South Africa

[2] National Institute for Theoretical Physics (NITheP), KwaZulu-Natal, 4001, South Africa

[3] School of Electrical Engineering, KAIST, Daejeon, 34141, Republic of Korea

E-mail: *betony@gmail.com

## 1. Supplementary information

### *1.1. Diagonalising the system Hamiltonian*

A basis is chosen for the electron spin states $\{|0\rangle,|1\rangle\}$, with $|0\rangle$ the excited state and $|1\rangle$ the ground state. From this the raising and lowering operators can be expressed as

$$S_+ = |0\rangle\langle 1|, \qquad S_- = |1\rangle\langle 0|.$$

$S_z$ in this basis is

$$S_z = \frac{1}{2}(|0\rangle\langle 0| - |1\rangle\langle 1|).$$

For the nuclear spins, as detailed above, with basis $\{|j,m\rangle, -j \leq m \leq j\}$ the raising and lowering operators are

$$I_+ = \sum_{m=-j}^{j-1} A^+_{jm}|j,m+1\rangle\langle j,m| \qquad I_- = \sum_{m=-j+1}^{j} A^-_{jm}|j,m-1\rangle\langle j,m|,$$

where $A^{\pm}_{jm} = \sqrt{j(j+1) - m(m\pm 1)}$. $I_z$ in this basis is

$$I_z = \sum_{m=-j}^{j} m|j,m\rangle\langle j,m|.$$

Due to the fact that the interaction between the two electrons of the radical pair is only in the initial conditions, after which each electron interacts with its own nuclear environment and bath, the dynamics of the electrons are treated separately. The system Hamiltonian for the first electron, using $S_xI_x + S_yI_y = \frac{1}{2}(S_+I_- + S_-I_+)$, can be rewritten as

$$H_S^{(1)} = \gamma_e B_0 S_z^{(1)} + \lambda_h(S_+^{(1)}I_-^{(1)} + S_-^{(1)}I_+^{(1)}) + \frac{\lambda_h}{2}(S_z^{(1)}I_z^{(1)}),$$

where the magnetic field points along the positive $z$ axis. Using the relevant expressions for the raising and lowering operators we arrive at a Hamiltonian that is not diagonal in the basis

$$\{|0,j,j\rangle, |1,j,-j\rangle, |1,j,m\rangle, |0,j,m-1\rangle\}.$$

By finding eigenvalues and eigenvectors we can write the new diagonal Hamiltonian in the basis of its eigenvectors

$$\{|0,j,j\rangle, |1,j,-j\rangle, |\lambda^-_{jm}\rangle, |\lambda^+_{jm}\rangle\}.$$

In the diagonal basis $|0,j,j\rangle$ and $|1,j,-j\rangle$ are already eigenvectors, having eigenvalues $\frac{1}{2}(\gamma_e B_0 + \lambda_h j)$ and $-\frac{1}{2}(\gamma_e B_0 - \lambda_h j)$ respectively. After normalisation the remaining eigenvectors are

$$|\lambda^-_{jm}\rangle = \frac{1}{\sqrt{1+\frac{1}{16}\left|\frac{k_m+2\Omega_{jm}}{\lambda_h A^-_{jm}}\right|^2}} \left( \frac{-k_m-2\Omega_{jm}}{4\lambda_h A^-_{jm}} 1 \right),$$

and

$$|\lambda^+_{jm}\rangle = \frac{1}{\sqrt{1+\frac{1}{16}\left|\frac{k_m-2\Omega_{jm}}{\lambda_h A^-_{jm}}\right|^2}} \left( \frac{-k_m+2\Omega_{jm}}{4\lambda_h A^-_{jm}} 1 \right),$$

with corresponding eigenvalues $v_1(j,m) = \frac{1}{4}(-\lambda_h - 2\Omega_{jm})$ and $v_2(j,m) = \frac{1}{4}(-\lambda_h + 2\Omega_{jm})$ with parameters

$$k_m = 2B_0\gamma_e + 2\lambda_h m - \lambda_h,$$

$$\Omega_{jm} = \sqrt{B_0^2\gamma_e^2 + B_0\gamma_e\lambda_h(2m-1) + \lambda_h^2\Big(4(A^-_{jm})^2 + \Big(m-\frac{1}{2}\Big)^2\Big)}.$$

The system Hamiltonian for the second electron is found in the same way, and is diagonal in the basis

$$\{|0,j,j\rangle, |1,j,-j\rangle, |\phi^-_{jm}\rangle, |\phi^+_{jm}\rangle\},$$

where the eigenvectors $|\phi^-_{jm}\rangle$ and $|\phi^+_{jm}\rangle$ with corresponding eigenvalues $u_1(j,m) = \frac{1}{4}(-\lambda_h - 2\Lambda_{jm})$ and $u_2(j,m) = \frac{1}{4}(-\lambda_h + 2\Lambda_{jm})$ rely on the parameters

$$k_m = 2B_0\gamma_e + 2\lambda_h m - \lambda_h,$$

$$\Lambda_{jm} = \sqrt{B_0^2\gamma_e^2 + B_0\gamma_e\lambda_h(2m-1) + \lambda_h^2\Big((A^-_{jm})^2 + \Big(m-\frac{1}{2}\Big)^2\Big)}.$$

### *1.2. The transition dipole moment*

For a transition $(n,1) \to (n+1,0)$ the transition dipole moment for a transition between two levels can be written as

$$d^2 = e^2|\,\langle (n+1,0)|\,\vec{\epsilon}.\vec{r}|\,(n,1)\rangle\,|^2 = e^2\left|\int_0^\infty r^3 R^*_{(n+1),0} R_{n1}(r)dr \int d\Omega Y^*_{00}\vec{\epsilon}.\hat{r}Y_{1m}\right|^2.$$

Considering the radial component, in the case of the hydrogen atom the normalised radial wave functions are

$$R_{nl} = -\Big(\frac{2}{na_0}\Big)^{\frac{3}{2}}\sqrt{\frac{(n-l-1)!}{2n[(n+l)!]^3}}\Big(\frac{2r}{na_0}\Big)^l e^{-\frac{r}{na_0}} L_{n+l}^{2l+1}\Big(\frac{2r}{na_0}\Big),$$

where the Laguerre polynomials are given by

$$L_k(r) = e^r \frac{d^k}{dr^k}\Big(r^k e^{-r}\Big),$$

and

$$L_k^n(r) = \frac{d^n}{dr^n} L_k(r).$$

For the transition

$$R_{n1} = -\Big(\frac{2}{na_0}\Big)^{\frac{3}{2}}\frac{1}{n}\sqrt{\frac{1}{2(n^2-1)}}\Big(\frac{2r}{na_0}\Big)e^{-\frac{r}{na_0}}\frac{1}{(n+1)!}L_{n+1}^3\Big(\frac{2r}{na_0}\Big),$$

and

$$R_{n+1,0} = -\Big(\frac{2}{(n+1)a_0}\Big)^{\frac{3}{2}}\frac{1}{\sqrt{2}(n+1)}e^{-\frac{r}{(n+1)a_0}}\frac{1}{(n+1)!}L_{n+1}^1\Big(\frac{2r}{(n+1)a_0}\Big).$$

Using this and with $n \gg 1, \frac{1}{n} \simeq \frac{1}{n+1}$ the radial integral can be calculated as

$$\int_0^\infty r^3 R^*_{(n+1),0}R_{n1}(r)dr$$
$$\simeq \frac{a_0}{4n^2}\int_0^\infty d\Big(\frac{2r}{na_0}\Big)\Big(\frac{2r}{na_0}\Big)^4 e^{-\frac{2r}{na_0}}\frac{1}{(n+1)!}L_{n+1}^3\Big(\frac{2r}{na_0}\Big)\frac{1}{(n+1)!}L_{n+1}^1\Big(\frac{2r}{na_0}\Big).$$

Let $\frac{2r}{na_0} = y$ and with some calculation

$$\int_0^\infty r^3 R^*_{(n+1),0}R_{n1}(r)dr = \frac{a_0}{4n^2}\int_0^\infty dy\; y^4 e^{-y}\tilde{L}_n^1(y)\tilde{L}_{n-2}^3(y),$$

using

$$\frac{d^k}{dx^k}\tilde{L}_n^\alpha(x) = (-1)^k \tilde{L}_{n-k}^{\alpha+k}(x),$$

with

$$\frac{d^3}{dy^3}\tilde{L}_{n+1} = (-1)^3\tilde{L}_{n-2}^3(y), \qquad \frac{d}{dy}\tilde{L}_{n+1}(y) = -\tilde{L}_n^1(y).$$

The frequency of hyperfine level transitions in the model are of the order of kHz to MHz. In order to estimate the transition dipole moment for transitions of this frequency they can be approximated as those of a Rydberg atom. Assuming a hydrogen-like atom (the assumption is made for ease of calculation and shouldn't effect the order of magnitude of the estimation, which is what we are interested in) the levels corresponding to a certain frequency can be calculated using the formula

$$\frac{1}{\lambda} = R\Big(\frac{1}{n^2} - \frac{1}{(n+1)^2}\Big),$$

where $R$ is the Rydberg constant and $\lambda$ is the wavelength corresponding to a transition between two levels.
